# Knowledge relation rank enhanced heterogeneous learning interaction modeling for neural graph forgetting knowledge tracing

**DOI:** 10.1371/journal.pone.0295808

**Published:** 2023-12-22

**Authors:** Linqing Li, Zhifeng Wang

**Affiliations:** 1 Central China Normal University Wollongong Joint Institute, Central China Normal University, Wuhan, China; 2 Faculty of Artificial Intelligence in Education, Central China Normal University, Wuhan, China; National University of Sciences and Technology NUST, PAKISTAN

## Abstract

Knowledge tracing models have gained prominence in educational data mining, with applications like the Self-Attention Knowledge Tracing model, which captures the exercise-knowledge relationship. However, conventional knowledge tracing models focus solely on static question-knowledge and knowledge-knowledge relationships, treating them with equal significance. This simplistic approach often succumbs to subjective labeling bias and lacks the depth to capture nuanced exercise-knowledge connections. In this study, we propose a novel knowledge tracing model called Knowledge Relation Rank Enhanced Heterogeneous Learning Interaction Modeling for Neural Graph Forgetting Knowledge Tracing. Our model mitigates the impact of subjective labeling by fine-tuning the skill relation matrix and Q-matrix. Additionally, we employ Graph Convolutional Networks (GCNs) to capture intricate interactions between students, exercises, and skills. Specifically, the Knowledge Relation Importance Rank Calibration method is employed to generate the skill relation matrix and Q-matrix. These calibrated matrices, alongside heterogeneous interactions, serve as input for the GCN to compute exercise and skill embeddings. Subsequently, exercise embeddings, skill embeddings, item difficulty, and contingency tables collectively contribute to an exercise relation matrix, which is then fed into an attention mechanism for predictions. Experimental evaluations on two publicly available educational datasets demonstrate the superiority of our proposed model over baseline models, evidenced by enhanced performance across three evaluation metrics.

## Introduction

In the age of rapid technological advancement, the ubiquity of networks has ushered in a wealth of information and convenience. This surge in data, commonly referred to as big data, plays a pivotal role in interconnecting diverse sectors such as education, healthcare, and transportation. The fusion of education with information science is an inexorable trend, catalyzing an expansive realm of research in online education technologies. Notably, the application of artificial intelligence and related technologies to analyze substantial educational data yields invaluable insights and enhances the educational landscape [1–4].

Online educational platforms, like edX, Coursera, and Udacity, have gained widespread adoption for tracking, reporting, and delivering online courses [3, 5, 6]. These platforms offer students a spectrum of advantages, including diverse online courses and personalized learning resources [6–8]. Moreover, they provide a valuable alternative to traditional classroom teaching, circumventing geographical or weather-related limitations. Teachers leverage these platforms to tailor remedial materials to suit individual student needs [4, 8–11].

**Fig 1:**
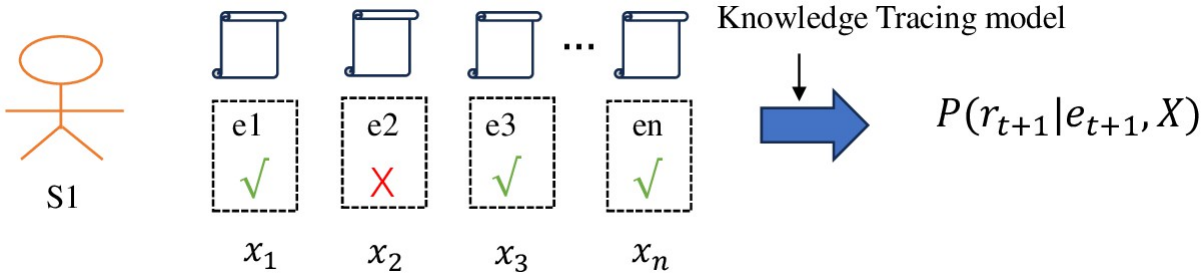
Illustration of the knowledge tracing model’s process.

Central to the effectiveness of online education is the task of tracking students’ performance based on their past interactions [12, 13]. This task, known as *Knowledge Tracing* [14, 15], seeks to gauge students’ knowledge states by analyzing their responses to exercises. Essentially, Knowledge Tracing (KT) assesses students’ mastery of knowledge. Given a set of practice questions *X*_*t*_ = (*x*_1_, *x*_2_, …, *x*_*t*_) and response logs (e.g., correct or incorrect), KT predicts the likelihood of correctly anticipating students’ knowledge states in subsequent interactions (*p*(*r*_*t*+1_ = 1|*e*_*t*+1_, *X*)) based on past interactions and responses. Here, *x*_*t*_ is presented as the tuple (*q*_*t*_, *a*_*t*_), where *q*_*t*_ represents the student’s question and *a*_*t*_ denotes the corresponding response. Refer to Fig 1 for a visual representation of the knowledge tracing model’s framework.

Numerous knowledge tracing models have emerged to tackle the challenge of monitoring students’ knowledge states, such as Deep Knowledge Tracing (DKT) [16], Dynamic Key-Value Memory Network (DKVMN) [17], Graph-based Knowledge Tracing (GKT) [18], and Self-Attentive Model for Knowledge Tracing (SAKT) [19]. These models exhibit improved predictive performance across several educational datasets. DKT incorporates deep neural networks to forecast student performance. However, it overlooks knowledge point information and student abilities. DKVMN employs nonlinear transformations to learn representations and master levels for each concept, but disregards the similarity between knowledge concepts (KCs) when making predictions. SAKT delves into the KC-exercise relationship for mastery level prediction but ignores the potential organizational structure of past interactions as a graph.

To address these limitations, we present the Knowledge Relation Rank Enhanced Heterogeneous Learning Interaction Modeling for Neural Graph Forgetting Knowledge Tracing (NGFKT). This model rectifies the shortcomings of existing models when predicting student performance from past interactions. The model encompasses four key steps: (1) Skill relation matrix and Q-matrix calibration using the Knowledge Relation Importance Rank Calibration method (KRIRC), which considers the students-exercise-KCs relationship; (2) Inputting the calibrated matrices into a Graph Convolutional Network (GCN) to yield skill and exercise embeddings; (3) Incorporating heterogeneous interactions, item difficulty, skill embeddings, and exercise embeddings to generate an exercise relation matrix; (4) Applying the Position-Relation-Forgetting attention mechanism, inspired by student forgetting behavior, to predict student performance. We validate our model on two datasets, demonstrating its superiority over traditional models.

The contributions of this paper encompass:

1. **Calibration Method for Enhanced Skill Relation Matrix and Q-Matrix**: By considering the intricate relationship between knowledge concepts (KCs) within heterogeneous interactions—namely students, exercises, and KCs—the calibration method ensures a more accurate representation. This calibrated skill relation matrix and Q-matrix, coupled with the heterogeneous interactions, serve as inputs for a Graph Convolutional Network (GCN). This integration facilitates the generation of comprehensive exercise embeddings and skill embeddings, effectively capturing the intricate interplay between students, exercises, and KCs.
2. **Exercise Relation Modeling with Enhanced Forgetting Mechanism**: In contrast to the GKT model [18], our proposed approach goes beyond surface-level prediction by incorporating the nuanced forgetting behavior of students and relative distance representations. This enhancement is realized through the utilization of the Position-Relation-Forgetting attention mechanism. This attention mechanism is designed to predict student performance, and its integration demonstrates the model’s ability to capture deeper insights into learning dynamics.
3. **Comprehensive Experimental Evaluation**: Our evaluation strategy addresses three key aspects. First, we measure the predictive efficacy of the NGFKT model against baseline models using three established metrics. Second, we assess the effectiveness of the NGFKT model even when dealing with limited records. Finally, we employ radar diagrams to visually depict and understand the knowledge tracing outcomes. This meticulous experimental evaluation bolsters the robustness of our proposed model and showcases its superiority in various scenarios.

The rest of this paper unfolds as follows: The next section, “Related Works,” delves into knowledge tracing models, graph neural networks, relation modeling, and attention mechanism. Subsequently, the “Methods” section provides a detailed exposition of NGFKT’s structure. The “Experiments” section presents implementation details and experimental outcomes. Finally, we conclude and highlight avenues for future research in the “Conclusions and Future Work” section.

## Related works

### Knowledge tracing

Knowledge Tracing (KT) aims to gauge students’ comprehension levels based on their historical interactions. The effectiveness of deep learning techniques in fields like speech processing (e.g., [20–23]) and computer vision (e.g., [24–27]) has inspired the development of deep learning-based KT models. One pioneering model is the Deep Knowledge Tracing (DKT) [16], which employs neural networks to capture intricate educational processes. The DKT+ model [28] extends DKT by introducing regularization items. However, these approaches overlook the influence of exercise embeddings. The Enhanced Knowledge Tracing (EKT) model [29] utilizes exercise embedding modules for predicting students’ future performance. The Memory Augmented Neural Network (MANN) [30] employs Key and Value metrics to model exercise embeddings and make predictions. Yet, these methods often neglect relation modeling to predict students’ knowledge state. To address this gap, the Self-Attentive Knowledge Tracing (SAKT) model [19] explores relationships between students, exercises, and skills in past interactions. Additionally, knowledge tracing can be framed as a graph-based task. The Graph-based Knowledge Tracing (GKT) model [18] formulates knowledge tracing as a time series node-level classification problem within a graph. However, the GKT model does not take exercise relation modeling and the heterogeneous interactions into consideration. Therefore, the our model is proposed to handle these limitations and further improve the prediction performance of the knowledge tracing model.

### Graph neural networks

Graph structures, which can represent connections and entities in the real world, are more intricate than simple tree structures [31]. Graph Convolutional Networks (GCNs), a unique type of neural network, operate directly on graph-structured data. A GCN updates node representations by considering the nodes themselves and their neighbors [32]. By incorporating multiple graph convolutional layers, GCNs ensure that updated nodes accurately capture both higher-order neighbor features and neighboring node features.

Several knowledge tracing models explore the utility of knowledge graph structures. Chanaa et al. [33] employ a dynamic graph structure in which nodes represent students and edges evolve over time. However, this method overlooks the heterogeneous interactions among students, exercises, and knowledge concepts (KCs). The Graph-Infused Knowledge Tracing (GIKT) model [13] treats the exercise-skill relationship graph as a bipartite graph and utilizes embedding propagation within GCNs to incorporate exercise-skill correlations. Nevertheless, GIKT doesn’t fully account for student behavior. Our proposed Neural Graph Forgetting Knowledge Tracing (NGFKT) model uses skill relation matrices and Q-matrices to model exercise-KC relationships, employing an attention mechanism enhanced with a forgetting layer to predict performance.

### Relation modeling

Relation modeling, including exercise relation modeling, is crucial in knowledge tracing tasks [34]. When two exercises are connected through shared knowledge concepts or students, their relation becomes significant [35]. Additionally, the Q-matrix can reveal relationships between exercises and skills, unveiling connections between exercise pairs [36]. Intuitively, if two problems share similar difficulty and past practice, they can be considered related [37, 38]. Building on these concepts, NGFKT integrates item difficulty, exercise embedding, skill embedding, and a contingency table to formulate a comprehensive exercise relation matrix.

### Attention mechanism

The attention mechanism is a powerful tool for sequence modeling tasks [39–41]. It enhances predictions by focusing on crucial input elements. The mechanism determines attention weights for input vectors, enabling it to extract relevant words in machine translation tasks [42–46]. In our model, we introduce a novel attention mechanism to predict student performance. This mechanism incorporates exercise relation modeling along with a forgetting behavior component, further enhancing prediction accuracy.

### Motivation

This paper aims to provide an innovative knowledge tracing model: NGFKT to estimate the student knowledge state based on the heterogeneous interactions between students, exercises, and skills. Specifically, the first motivation is to design a calibration method to generate the Q matrix and skill relation matrix. The second motivation is to develop an exercise relation modeling method to obtain an exercise relation matrix. Finally, incorporating the attention mechanism with the relation matrix predicts student performance.

## Preliminaries

In this section, on the one hand, we summarize the important mathematical notation covered in this paper in Table 1, and on the other hand, we give important terminology and problem definitions.

**Table 1:** Important notations.

Notations	Description
KT	The knowledge tracing model
KC	Knowledge concepts
$\hat{Q}$	The calibrated Q-matrix
$\hat{S}$	The calibrated skill reltion matrix
M	The calibrated matrix
*a*_*rank*_	The knowledge relation importance rank of the element: “a”
*b*_*rank*_	The knowledge relation importance rank of the element: “b”
K	The number of the knowledge concepts
N	The number of the questions
*σ*	The standard deviation
$\hat{e}$	Skill-exercise embedding
q	One question in the question set
s	One student in the student set
*ψ*_*s*,*q*,*t*_	the cognitive difficulty of the question: q for the student: s
*R* ^*E*^	The exercise relation matrix
*I*_*i*_	The input element of the Position-Relation-Forgetting attention mechanism

### Terminologies

#### Heterogeneous interactions

**Fig 2:**
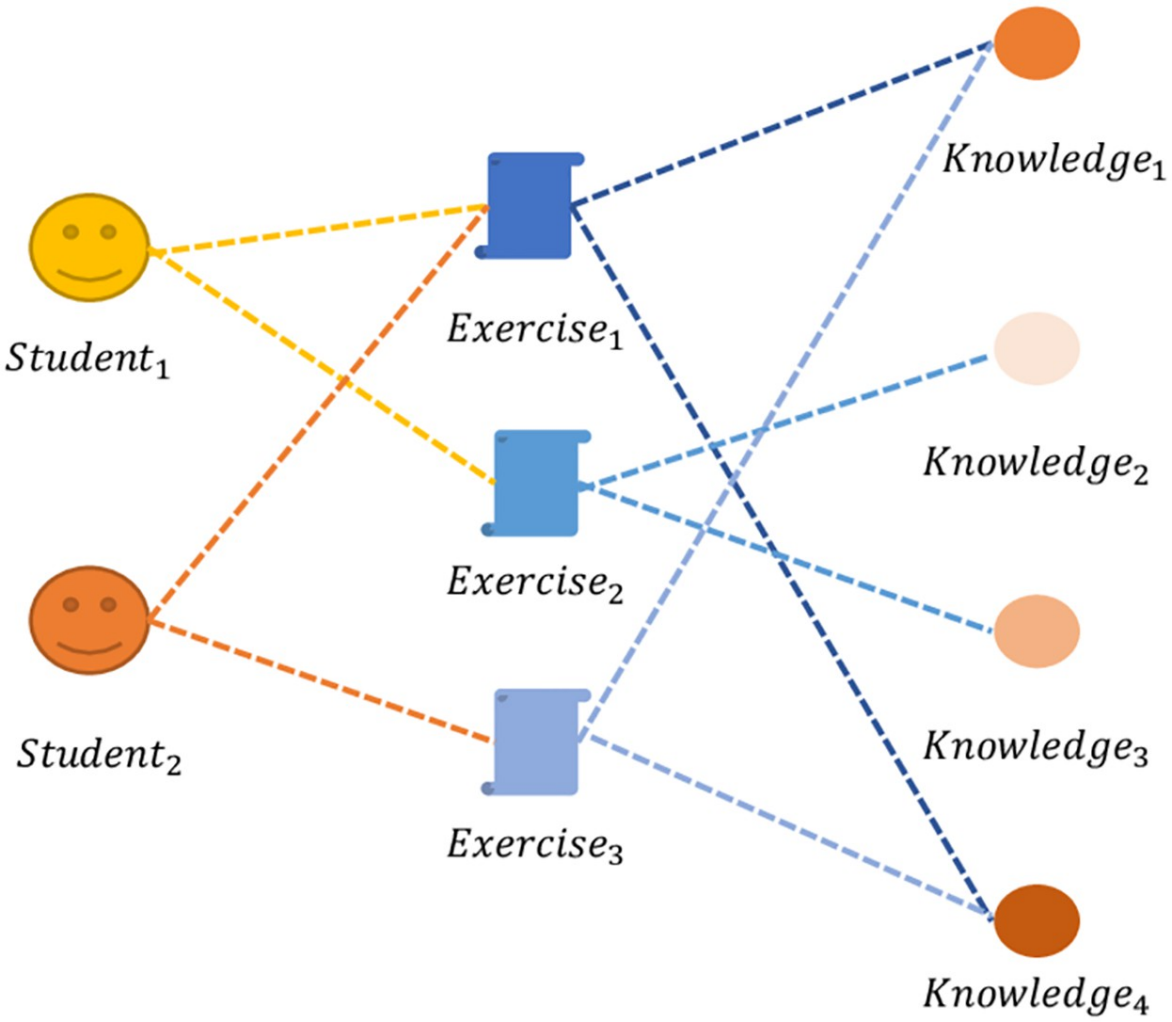
The heterogeneous interaction between student, exercise, and skills.

There are numerous interactions between students, exercises, and skills referring to the Fig 2. Heterogeneous interaction (H) is formed up of an object set, V, and a link set, E. Students, exercises, and skills are all object types in V. E is a set of relational types of the type E = (*r*_*A*_,*r*_*C*_). The *r*_*A*_ indicates the response of exercises and *r*_*C*_ means the relation it involved.

#### Knowledge tracing

Knowledge Tracing (KT) evaluates learners’ knowledge mastery. KT predicts the likelihood of effectively estimating students’ knowledge states in coming meets given a set of test questions and answer logs (e.g., correct or incorrect).

#### Student modeling

At the same time, based on the results of knowledge tracking, we can model student groups and individual students. Specifically, if the input is a student group, then the result of our knowledge tracking is the modeling of the student group. If the input is an individual student, then the knowledge tracking is the modeling of a single student.

#### Exercise modeling

In this paper, we model the exercises based on the exercise relationship on the heterogeneous interaction graph. Specifically, we apply the graph neural network to process the exercises and discuss the difficulty of the exercises to generate the final exercise embedding.

#### Knowledge modeling

In order to comprehensively model the knowledge in this paper, we apply the knowledge relation importance rank calibration method to model the knowledge contained in the exercises and generate the skill relation matrix. Then this matrix is treated as the input of GCN to generate the hidden embedding of exercises.

### Problem definition

The computerized system generates the student’s responding records given a question set of n exercises (e.g., *e*_0_, *e*_1_, *e*_2_…*e*_*n*_) for the student in the smart educational system from Timestamps 1 to t. Those interactions are designated as S = (*s*_1_, *s*_2_, *s*_3_…‥ *s*_*n*_), and each interaction *s*_*i*_ is presented as a tuple: *s*_*i*_ = (*e*_*i*_,*r*_*i*_,*t*_*i*_), where *e*_*i*_ is the exercise that this student attempted, *r*_*i*_ ∈ {0, 1} is the student’s answer, and *t*_*i*_ is the time at which *s*_*i*_ happens. Our goal is to estimate the knowledge state of students based on previous information. Specifically, we input the heterogeneous interaction graph and previous student records: S, the output of the knowledge tracing model is the knowledge state of students.

## Methods

**Fig 3:**
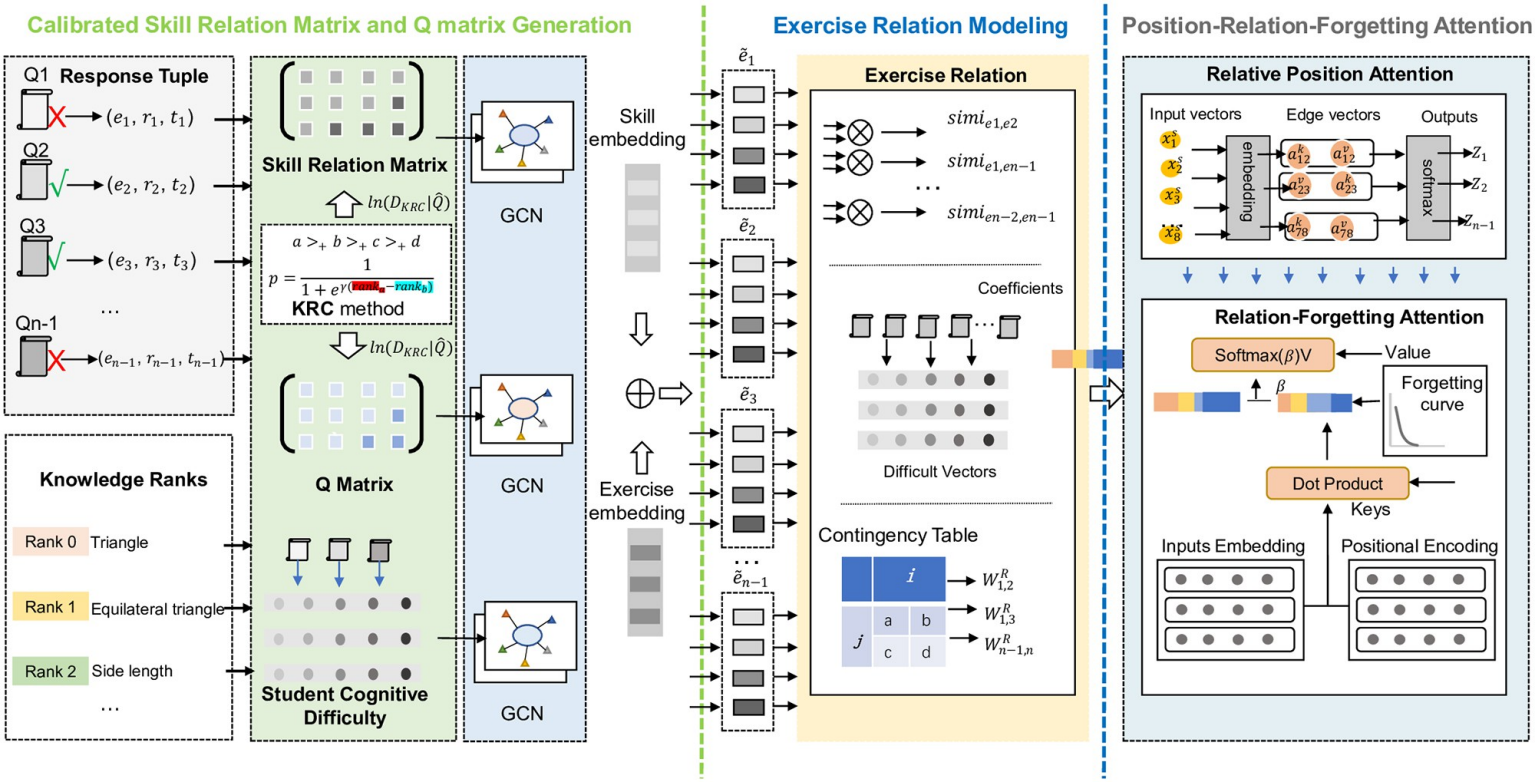
The overall structure of the Neural Graph Forgetting Knowledge Tracing (NGFKT). Firstly, according to previous interactions, the past interactions of students and knowledge levels can be extracted from the inputs. Then KRIRC method is designed to generate the calibrated skill relation matrix and Q-matrix. The skill relation matrix, Q-matrix, and the cognitive difficulty of each student are treated as the inputs of the GCN to generate the skill-exercise embedding: $\hat{e}$. Secondly, the $\hat{e}$, the item difficulty of each question, and the contingency table are combined to output the exercise relation matrix. Finally, the Position-Relation-Forgetting attention mechanism is utilized to process the inputs to make predictions.

In this section, the NGFKT model is developed based on the exercise relation matrix and Position-Relation-Forgetting attention mechanism to predict the performance of students. There exist three steps including the skill relation matrix and Q-matrix modeling, the extraction of the exercise relation matrix, and the predictions based on the Position-Relation-Forgetting attention mechanism. First, the skill relation matrix($\hat{S}$) and the calibrated Q-matrix ($\hat{Q}$) are designed based on the KRIRC method and serve as the input of the model. Then, the skill relation matrix $\hat{S}$, and the calibrated Q-matrix ($\hat{Q}$) are treated as the inputs of the GCN to output the embedding of exercises and skills in the heterogeneous interactions. The similarity of exercises can be computed based on the exercise embedding, skill embedding, item difficulty, and contingency table to generate the corresponding exercise relation matrix(*R*^*E*^). Finally, the exercise relation matrix(*R*^*E*^) serves as the input of the Position-relation-Forgetting attention mechanism to track the student knowledge state. The overall structure can refer to Fig 3.

### Skill relation matrix and Q matrix modeling

We design this part to extract the heterogeneous information between students, exercises, and skills. The basic idea of this part is that the related skill can be considered as the skill that is covered by the same exercise. Considering the hierarchical knowledge levels of skill, the related skill also can be defined as the parent nodes and child nodes of the skill. For instance, the knowledge concept: the “Triangle” is viewed as the “Right Triangle”’s parent node, and the “Pythagorean Theorem” is the child node of the “Right Triangle”. Therefore, the related skills of the “Right Angle” are the concepts: “Triangle” and “Pythagorean Theorem”. Inspired by these two ideas, the importance of the skills is ranked based on the following partial order, and the calibrated method called the knowledge Relation Importance Rank Calibration method is proposed(KRIRC). A pairwise Bayesian treatment is as follows. For convenience, we define a partial order ${>}_{i}^{+}$ as:

$$\begin{array}{c} a{>}_{i}^{+}b{>}_{i}^{+}>c{>}_{i}^{+}d \end{array}$$ (1)

where “a”, “b”, “c”, and “d” are the neighbors of the skill. Here, “a” implies the skill of the parent node, knowledge level is 0, in the knowledge level graph. “b” denotes the skill of the child node, knowledge level is 1, in the knowledge level graph. “c” can be interpreted in a similar way. “d” is the skill that is covered by the same exercise. Along this line, neighbor: “a” is more important than neighbor: “b” in extracting the neighbor of the skill. The rank of the skill: “a”, “b”, “c”, and “d” are 0, 1, 2, and 3 respectively. Thereby, according to the Eq 1, the partial order relationship set can be defined as $D_{KRIRC}=\left\{\left(i,a,b\right)|a{>}_{i}^{+}b,i=1,2,3\ldots K\right\}$ where K is the number of knowledge concepts. Based on the traditional Bayesian method, we assume the calibration matrix: $\hat{M}$ such as $\hat{Q}$ or $\hat{S}$ uniforms the Gaussian distribution with the standard deviation of each dimension. To give the calibration matrix labels higher confidence, we define $p(a{>}_{i}^{+}b|\hat{M})$ as follows:

$$\begin{array}{c} p\left(a{>}_{i}^{+}b|\hat{M}\right)=\frac{1}{1+e^{\lambda (a_{rank}-b_{rank})}} \end{array}$$ (2)

where λ controls the discrimination of relevance values of different knowledge levels. And the *a*_*rank*_ and *b*_*rank*_ means the importance of the element a or b. The log posterior over D_*KRIRC*_ on $\hat{M}$ can be eventually computed as:

$$\begin{array}{ccc} lnp(\hat{M}|D_{KRIRC}) & = & ln\prod_{\left(i,a,b\right)\in D_{KRIRC}}^{1}p\left(a{>}_{i}^{+}b|\hat{M_{i}}\right)p\left(\hat{M_{i}}\right)\\ \; & = & \sum_{i=1}^{N}\sum_{a=1}^{K}\sum_{b=1}^{K}I\left(a{>}_{i}^{+}b\right)ln\frac{1}{1+e^{\lambda (a_{rank}-b_{rank})}}+C-\sum_{i=1}^{N}\sum_{j=1}^{K}\frac{{\hat{M}}_{ij}^{2}}{2\sigma^{2}} \end{array}$$ (3)

where I(*) is the judgment function when the function’s condition is met and the function output 1. The *σ* is the standard deviation in Eq 2 and C, the constant variable, can be ignored to train the matrix. Finally, a calibrated matrix $\hat{M}$ estimated by the KRIRC can be calculated. The Q-matrix and the skill relation matrix can apply the KRIRC method to obtain the calibrated Q-matrix and the calibrated skill relation to reflect the hierarchical knowledge levels between different knowledge points. The specific algorithm can be referenced as follows:

**Algorithm 1**: Knowledge Relation Importance Rank Calibration Method.

**Input**: Students’ historical response dataset: *D* = *s*_1_, *s*_2_, …*s*_*N*_, *s*_*i*_ = (*e*_*i*_, *s*_*i*_, *t*_*i*_);

   The knowledge level graph G; The heterogeneous relation graph: *τ*;

   Task-learning rate: *α*; Hyper-parameter λ;

**Output**: The calibrated Q matrix: $\hat{Q}$; The calibrated skill relation matrix: $\hat{S}$.

**1** initialization learning rate *α* and hyper-parameter λ randomly

**2 while** *element in G and τ* **do**

**3**  Extract hierarchical knowledge levels and related skills of each element based on G and *τ*

**4**  Generate the corresponding knowledge rank using Eq 1

**5 end**

**6 while** *not converged* **do**

**7  while** *element in* $\hat{Q}$ *and* $\hat{S}$ **do**

**8**   Evaluate calibrated skill element *q*_*ij*_ using Eq 2;

**9**   Replace the element in $\hat{Q}$ and $\hat{S}$ with a calibrated element

**10**  **end**

**11**  Update parameters learning rate *α* and hyper-parameter λ

**12 end**

### Exercise relation modeling

The exercise matrix modeling is designed based on two processes. Firstly, skill-exercise embedding:$\hat{e}$ is calculated by applying the GCN. Secondly, the item difficulty is modeled to estimate the difficulty of each question. And $\hat{e}$, item difficulty, and the contingency table are incorporated to generate the final exercise relation matrix:*R*^*E*^.

The basic idea of the GCN model is to extract the neighbor information to generate the target node embedding. For the GCN model, we apply it to process the relationship of exercises and skills to generate the hidden exercise embedding. In this case, the heterogeneous information can be extracted from the skill relation matrix and the Q-matrix based on previous matrix modeling. Then, based on the knowledge tracing results, we can model the student states to explore the student information in the heterogeneous interactions. These matrices are served as the input of GCN. The GCN model consists of numerous convolutional layers, and each layer can be updated by the states of itself and the neighbors of nodes. The *i*th node in the graph donated as *node*_*i*_ indicating the skill state s_*i*_ or exercise state e_*i*_. The neighboring nodes of *node*_*i*_ are denoted as the node set: Node(i). As a result, the *ı* th layer of the graph convolutional network can be updated as follows:

$$\begin{array}{c} node_{i}^{ı}=RELU\left(\sum_{j\in i\cup Node(i)}^{}w_{i}^{ı}node_{j}^{ı-1}+b_{i}^{ı}\right) \end{array}$$ (4)

where w^*ı*^ and b^*ı*^ infer as the weighted matrix and bias of the GCN layer and the RELU() indicates the activation function accepted in the GCN model. The output of the GCN model:$\hat{e}$ is used for estimating the implicit relations among questions by calculating the inner product of questions:

$$\begin{array}{c} simi_{i,j}=\frac{\hat{e_{i}}\cdot \hat{e_{j}}}{|\hat{e_{i}}\left|\right|\hat{e_{j}}|} \end{array}$$ (5)

Then, in order to evaluate the exercises’ similarity, we further incorporate the item difficulty with the previous students’ performance to generate an exercise relation matrix: *R*^*E*^. The basic idea is that we apply the student incorrect interactions to estimate the item difficulty and seven metrics, which are used to measure the performance of different variables, to estimate the exercises relation.

For the item difficulty modeling, the students’ incorrect interactions can intuitively represent the item difficulty of the exercises involved in the student interactions. Specifically, the students repeat the same questions by utilizing their skills in different timestamps, which can demonstrate the cognitive difficulty of these exercises. In order to model this situation, the cognitive difficulty for the students: s can be defined as follows:

$$\begin{array}{c} \begin{array}{c} \Psi_{s,q,t}=\{\begin{array}{cc} \left[\frac{|\left\{R_{s}==0\right\}{|}_{0:t}}{|Q_{q}{|}_{0:t}}*4\right] & if|Q_{q}{|}_{0:t}\geq 5\\ 5 & otherwise \end{array} \end{array} \end{array}$$ (6)

where Ψ_*s*,*q*,*t*_ indicates each student’s cognitive difficulty of the question set at timestamp t. The cognitive difficulty is divided into 5 levels including very hard, hard, medium difficulty, relatively easy, and easy. The number ranging from 0 to 5 is accepted to indicate the corresponding levels. The |*Q*| denotes the set of questions containing the question q before timestamp t and *R*_*s*_ refers to the student’s response to the same questions. A zero in the *R*_*s*_ indicates that the student provides a wrong answer for a question. If a learner attempts to answer a question fewer than five times, the cognitive difficulty of this question is directly quantified into 5. Then according to the different learners’ cognitive difficulty of questions, the average cognitive difficulty for different learners on the same question is defined as the item difficulty: *φ*(*q*) after processing the cognitive difficulty for different learners.

$$\begin{array}{c} \varphi \left(q\right)=\frac{1}{|\Psi_{s,q,t}|}\sum_{\Psi \left(s_{i},q,t\right)\in \Psi \left(s,q,t\right)}^{}\Psi \left(s_{i},q,t\right) \end{array}$$ (7)

Then, according to the item difficulty: *φ*_*q*_, the similarity of question difficulty: *diff* can be modeled as follows:

$$\begin{array}{c} diff\left(q_{i},q_{j}\right)=\frac{1}{1+\varphi \left(q_{i}\right)-\varphi \left(q_{j}\right)} \end{array}$$ (8)

In order to incorporate the previous interaction, the student’s performance on question pair *q*_*i*_ and *q*_*j*_ is summarized in the contingency table. The students’ correct and incorrect responses are interpreted as the mastery indicators of the questions referring to Table 2. When a question pair appears more than once in the previous student interactions, the latest occurrence is taken into consideration. According to the contingency table, seven evaluation metrics, measuring the association between two variables, are developed to measure the relationship between the question pair: *e*_*i*_ and *e*_*j*_ referring to Table 3. A threshold is imposed to control the sparsity of relations of exercises. The output of the contingency table is denoted as *W*^*R*^, *R* ∈ {*SK*, *Kappa*, *Kappa*′, *Phi*, *Yule*, *Ochiai*, *Sokal*, *Jaccard*.}.

$$\begin{array}{c} \begin{array}{c} \left\{ \begin{array}{cc} W_{i,j}^{R}=max\left(W_{i,j}^{R},W_{j,i}^{R}\right),W_{j,i}^{R}=0 & W_{i,j}^{R}\geq W_{j,i}^{R}\\ W_{j,i}^{R}=max\left(W_{j,i}^{R},W_{i,j}^{R}\right),W_{i,j}^{R}=0 & otherwise \end{array}\right. \end{array} \end{array}$$ (9)

**Table 2:** The contingency table for exercise *i* and exercise *j*. The labels: “F” and “T” present the student answering the exercise incorrectly or correctly.

		exercise i		
		F	T	total
**exercise j**	F	a	b	a+b
	T	c	d	c+d
	total	a+c	b+d	a+b+c+d

**Table 3:** Seven evaluation metrics. These metrics are designed to explore the association between two variables.

Metrics	Formulation
Cohen’s Kappa	$W_{e_{i},e_{j}}^{Kappa}=2(\text{ad}-\text{bc})/(\text{a}+\text{b})(\text{b}+\text{d})+(\text{a}+\text{c})(\text{c}+\text{d})$
Adjusted Kappa	$W_{e_{i},e_{j}}^{Kappa^{{}^{\prime }}}=2(\text{ad}-\text{bc})/(\text{a}+\text{c})(\text{c}+\text{d})$
Phi coefficient	$W_{e_{i},e_{j}}^{Phi}=(\text{ad}-\text{bc})/\sqrt{\left(a+b\right)\left(b+d\right)\left(a+c\right)\left(c+d\right)}$
Yule coefficient	$W_{e_{i},e_{j}}^{Yule}=(\text{ad}-\text{bc})/(\text{ad}+\text{bc})$
Ochiai coefficient	$W_{e_{i},e_{j}}^{Ochiai}=\text{a}/\sqrt{\left(a+b\right)\left(a+c\right)}$
Sokal coefficient	$W_{e_{i},e_{j}}^{Sokal}=(\text{a}+\text{d})/\sqrt{\left(a+b+c+d\right)}=(\text{a}+\text{d})/\sqrt{\left(a+b+c+d\right)}$
Jaccard coefficient	$W_{e_{i},e_{j}}^{Jaccard}=\text{a}/(\text{a}+\text{b}+\text{c})$

Finally, the relation of exercise: i with exercise: j is calculated as follows:

$$\begin{array}{c} \begin{array}{c} A_{i,j}=\{\begin{array}{cc} \mu_{1}simi_{i,j}+\mu_{2}diff\left(q_{i},q_{j}\right)+\mu_{3}W_{i,j}^{R} & if\;\mu_{1}simi_{i,j}+\mu_{2}diff\left(q_{i},q_{j}\right)+\mu_{3}W_{i,j}^{R}\geq \Theta \\ 0 & otherwise \end{array} \end{array} \end{array}$$ (10)

where Θ is a threshold to control the sparsity of the exercise relation matrix. Then Given the past exercises: (*e*_1_, *e*_2_, …*e*_*n*−1_) and the next exercise: *e*_*n*_, the exercise relation matrix is defined as $R^{E}=[A_{e_{n},e_{1}}$, $A_{e_{n},e_{2}},\ldots A_{e_{n},e_{n-1}}]$. Finally, the exercise relation matrix is applied as the input of the Position-Relation-Forgetting attention mechanism.

### Position-Relation-Forgetting attention mechanism

The Position-Relation-Forgetting attention mechanism includes the relative position attention layer, the relation attention layer, and the forgetting layer. The basic idea of the relative position attention aims to replace the positional encoding of input embedding with relative distance embedding. Specifically, the maximum absolute value of k is used to clip the distance of words in the input embedding. The relative position attention accepts the relative distance between input elements. *I*_*i*_ and *I*_*j*_ serve as the model inputs to track the student state. And edge vectors between *I*_*i*_ and *I*_*j*_ are presented as $a_{i,j}^{v},a_{i,j}^{K}$ to extract relative position representations. The edge vectors are clipped to a maximum absolute value of k: clip(x,k) = max(-k, min(k,x)). And corresponding relative position representations are $W^{K}=\left(W_{-k}^{k}\ldots .W_{k}^{K}\right)$ and $W^{V}=\left(W_{-k}^{V}\ldots .W_{k}^{V}\right)$ respectively. The outputs of the relative position attention mechanism are new sequences *Z*. The process can refer to the following equations:

$$\begin{array}{c} a_{i,j}^{K}=W_{clip(j-i,k)}^{k}\;a_{i,j}^{V}=W_{clip(j-i,k)}^{V} \end{array}$$ (11)

$$\begin{array}{c} a_{i,j}=\frac{exp(m_{i,j})}{\sum_{i=1}^{n}exp\left(m_{i,k}\right)}\;m_{i,j}=\frac{I_{i}W^{Q}{\left(I_{j}W^{k}\right)}^{T}+I_{i}W^{Q}{\left(a_{i,j}^{K}\right)}^{T}}{\sqrt{d_{z}}} \end{array}$$ (12)

$$\begin{array}{c} Z_{i}=\sum_{j=1}^{n}a_{ij}\left(I_{j}W^{V}\right) \end{array}$$ (13)

where W^*Q*^, W^*K*^, and W^*V*^ are the query, key, and value matrices respectively and d_*z*_ is the dimension of the new sequence of *Z*. Then, the relation attention predicts student performance on the next interaction by combining the outputs of the relative position attention mechanism.

The relation attention layer incorporates the output of the formula (13) with the exercise relation matrix to predict student performance on the next interaction. The basic idea of the relation attention layer is to incorporate the relation matrix into the attention mechanism to consider the relational information in data.

$$\begin{array}{c} h_{i}=\frac{E_{e_{n}}W^{Q}{\left(Z_{j}W^{K}\right)}^{T}}{\sqrt{d}}\;\alpha_{i}=\frac{exp(h_{i})}{\sum_{k=0}^{n-1}exp\left(h_{k}\right)} \end{array}$$ (14)

$$\begin{array}{c} \gamma_{i}=\delta \alpha_{i}+\left(1-\delta \right)R_{i}^{E}\;H=\sum_{i=1}^{n-1}\gamma_{i}Z_{i}W^{v} \end{array}$$ (15)

where W^*Q*^, W^*K*^, and W^*V*^ represent the query, key, and value matrices of the attention mechanism and $E_{e_{n}}$ means the embedding of exercises.

Then applying the output of the relation attention layer is treated as the input of the forgetting layer based on learning theory in the educational field. The basic idea of part is to utilize the time interval between past time and current time to estimate the student forgetting curve. The relative time intervals between past and next interactions are compared as △_*i*_ = *t*_*n*_ − *t*_*i*_. The final outputs of the Position-Relation-Forgetting attention mechanism incorporating forgetting behavior, *R*^*F*^, is computed as follows:

$$\begin{array}{c} R^{F}=\left[\xi_{1}e^{-\xi_{2}{△}_{1}},\xi_{1}e^{-\xi_{1}{△}_{2}}\ldots \xi_{1}e^{-\xi_{2}{△}_{n}}\right]\;O=\delta_{F}H+\left(1-\delta_{F}\right)R^{F} \end{array}$$ (16)

where *ξ*_1_ and *ξ*_2_ are hyper-parameters.

### Student performance prediction layer

The student performance prediction layer contains the pointwise Feed-Forward (FFN) and probability prediction layer. The FFN can be computed as follows referring to(17). *W*_*l*_ and *W*_*s*_, *b*_*l*_ and *b*_*s*_ are weighted matrices and bias vectors respectively.

$$\begin{array}{c} F=ReLU\left(OW_{l}+b_{l}\right)W_{s}+b_{s} \end{array}$$ (17)

The probability prediction layer predicts the probability of the student’s performance by accepting function: *σ*() on the basis of the FFN. *P* denotes as the probability that the students provide correct answers in the next interaction. The W and b are trainable parameters.

$$\begin{array}{c} p=\sigma (FW+b) \end{array}$$ (18)

## Experiments

### Implementation details

#### Framework setting

The model dimension of attention, the max sequence length, and the training batch size are 200. The dropout rate of the NGFKT model is 1e-2. And the hyper-parameters, including the λ, Θ, are 1 and 0.65 respectively. The parameters in the exercise relation modeling: *μ*_1_, *μ*_2_, and *μ*_3_ are 0.1, 0.2, and 0.7 respectively. The other parameters that are not specified involved in the process of the training model are normally initialized as 0. The details can refer to the following Table 4.

**Table 4:** The framework setting for the Neural Graph Forgetting Knowledge Tracing model(NGFKT).

Parameters	Setting
Max sequence length	200
Training batch size	200
Dropout rate	0.01
λ	1
Θ	0.65
*μ*_1_	0.1
*μ*_2_	0.2
*μ*_3_	0.7

### Evaluation metrics

There exist three metrics to measure the performance of our model including the Area Under Curve(AUC), Accuracy(ACC), and Performance Stability(PS). The prediction task is evaluated in a binary classification scenario, i.e., whether or not an exercise is performed correctly. As a result, the AUC and ACC are accepted to measure the prediction performance of students. AUC or ACC values of 0.5 usually indicate that the result was determined at random. The greater the knowledge tracing performance, the higher the value of AUC or ACC. The cross-entropy is accepted as the loss function of the NGFKT model.

The Performance Stability metric(PS) is used to specifically compare the performance of the baseline models with the NGFKT model in the testing phase. The performance of the model: M is stable when the M can consistently outperform other models in most testing batches. Based on this idea, the PS is designed based on the performance rank. For instance, if the NGFKT model outperforms the DKT model and DKT+ model in 96 testing batches. However, the performance of the NGFKT model is worse than the DKT+ model in 4 testing batches. The performance rank of the NGFKT model is 1 in 94 testing batches and 2 in 4 testing batches. Then the PS of the NGFKT model is 97.32% referring to the following formulation. The *N*_*Batch*_ and *N*_*model*_ are the number of the testing batches and models in this paper.

$$\begin{array}{c} PS\left(M\right)=\sum_{i=0}^{N_{Batch}}\frac{N_{model}-rank\left(M,i\right)+1}{N_{model}} \end{array}$$ (19)

### Datasets

In order to demonstrate the performance of the NGFKT model in the small dataset and the large dataset, two types of public educational datasets were accepted to verify the performance of the NGFKT model in terms of AUC, ACC, and PS.

The statistical information of the datasets is provided in Table 5. There are two public educational datasets to validate the performance of the NGFKT model.

**Table 5:** The overview of ASSIST2012 and Eedi based on different intelligent tutoring systems.

Statistic	ASSIST2012	Eedi
Number of records	4193631	233767
Number of students	39364	2064
Number of questions	59761	948
Avg exercise record/student	107	113

#### ASSISTments2012(ASSIST2012)

The first dataset is the large dataset: ASSISTments2012(ASSIST2012) (available at: https://sites.google.com/site/assistmentsdata/datasets/2012-13-school-data-with-affect), which was gathered by the *Assistemt Online Tutoring platforms*. The ASSIST2012 contains 4193K records of 39K students. Each student answers an average of 107 questions.

There are several datasets that are collected by *Assistemt Online Tutoring platforms*. The reason for choosing ASSIST2012 is that the NGFKT model needs the timestamp feature in the dataset to model the forgetting behavior of students. However, other datasets *Assistemt Online Tutoring platforms* do not gather the timestamp feature to make predictions. Therefore, ASSIST2012 is accepted as the training dataset to validate the performance of the NGFKT model.

#### Eedi

The second dataset is the small dataset: the Eedi dataset (available at: https://eedi.com/projects/neurips-education-challenge) collected by the NeuralIPS platform, which includes 233K records and 2064 students. And an average of 113 exercises are provided for each student. In this paper, tasks 3 and 4 in the NeuralIPS system are applied to track the knowledge state of students.

### Results and discussion

#### Student performance prediction

The experimental results are presented in Table 6. The AUC, ACC, and PS are computed to compare the performance of different models. In order to verify the prediction performance of student abilities, the training set and testing set are divided into 80% records of the dataset and 20% records of the dataset respectively.

**Table 6:** Comparison of results of baseline models with the Neural Graph Forgetting Knowledge Tracing model(NGFKT). The NGFKT outperforms all baseline models in terms of AUC, ACC, and PS.

	ASSIST2012			Eedi		
	AUC	ACC	PS	AUC	ACC	PS
DKT	0.712	0.679	0.142	0.489	0.489	0.179
DKT+	0.722	0.685	0.232	0.584	0.566	0.501
DKVMN	0.701	0.686	0.392	0.701	0.640	0.829
SAKT	0.736	0.692	0.732	0.495	0.495	0.216
GKT	0.702	0.687	0.491	0.566	0.542	0.354
**NGFKT**	**0.776**	**0.704**	**0.960**	**0.710**	**0.673**	**0.937**

In two educational datasets, the NGFKT model is compared with five baseline models, including the DKT model, the DKT+ model, the DKVMN model, the SAKT model, and the GKT model. As Table 6 illustrated, the DKT+ model outperforms the DKT model on two datasets due to the fact that two regularization terms are accepted by the DKT+ model to solve the reconstruction problem and the consistency problem in the DKT model. However, knowledge concept modeling is ignored in the DKT model and DKT+ model. Therefore, the DKVMN model applies the nonlinear transformations and student master level on each KC to make predictions and performs better than the DKT+ model and DKT model on the Eedi dataset. However, the DKVMN model performs worse than the DKT model and DKT+ model on the ASSIST2012 because the number of questions is so large on the ASSIST dataset that the Knowledge concepts modeling is excessively complex. Compared with the DKVMN model, the SAKT model further applies the attention mechanism to process the data and discovers the relation between KCs to obtain better performance than the DKVMN model on the ASSIST2012 dataset. However, the SAKT model possesses worse performance than the DKVMN model on the Eedi dataset. The reason is that the number of KCs on the Eedi is less than the ASSIST so the relation modeling of KCs in the SAKT model possesses less effect on the prediction results. In addition, the GKT model does not perform as well as we thought because considering the nodes of the graph established based on two datasets is relatively sparsity. Therefore, the performance of the GKT model is worse than most baseline models. These baseline models do not consider the relation modeling of the exercises and forgetting behavior of students. Therefore, the NGFKT model is proposed to solve these drawbacks. The NGFKT model incorporates the exercise relation modeling with the novel attention mechanism: Position-Relation-Forgetting attention mechanism to make predictions. The NGFKT model performs consistently better than all baseline models in terms of three evaluation metrics.

#### Ablation experiments

This part aims to find out the key components of our model and validate the performance of these key components. There exist two variants models of the NGFKT model: the NGFKT-PE and the NGFKT-RM, which means that the NGFKT model removes the relative position encoding(PE) or relation modeling(RM)respectively. Specifically, the positional encoding and relation modeling are implemented by the position attention layer and the relation attention layer with the forgetting attention layer. the According to Table 7, some conclusions can be drawn.

**Table 7:** The ablation experiments of the Neural Graph Forgetting Knowledge Tracing model(NGFKT).

	ASSIST2012			Eedi		
	AUC	ACC	PS	AUC	ACC	PS
NGFKT-RM	0.740	0.678	0.678	0.684	0.637	0.804
NGFKT-PE	0.753	0.689	0.860	0.691	0.641	0.679
**NGFKT**	**0.776**	**0.704**	**0.960**	**0.710**	**0.673**	**0.937**

Firstly, the individual components of the position attention layer and the relation attention layer do not generate satisfactory outcomes when used alone. The performance of the model is gradually better when more components are involved in the model. Secondly, removing the RM component leads to a significant drop, decreasing to 0.740 and 0.684 respectively compared with removing the PE components. Therefore, the RM is a more crucial component for the NGFKT model when the model makes predictions.

#### Cold start problem

The cold start issue also impacts knowledge tracing when the model predicts the knowledge state of students. The results are examined in two cold start scenarios, i.e., training with data from a small number of students and new students with abbreviated exercise sequences [47].

1. The knowledge tracing model is trained using data from a limited number of students, and it is then applied to completely new, untested samples.
2. When new students enroll in an online learning system, they often have short exercise sequences since there aren’t enough exercise recordings to provide the knowledge tracing model with enough information about them.

**Fig 4:**
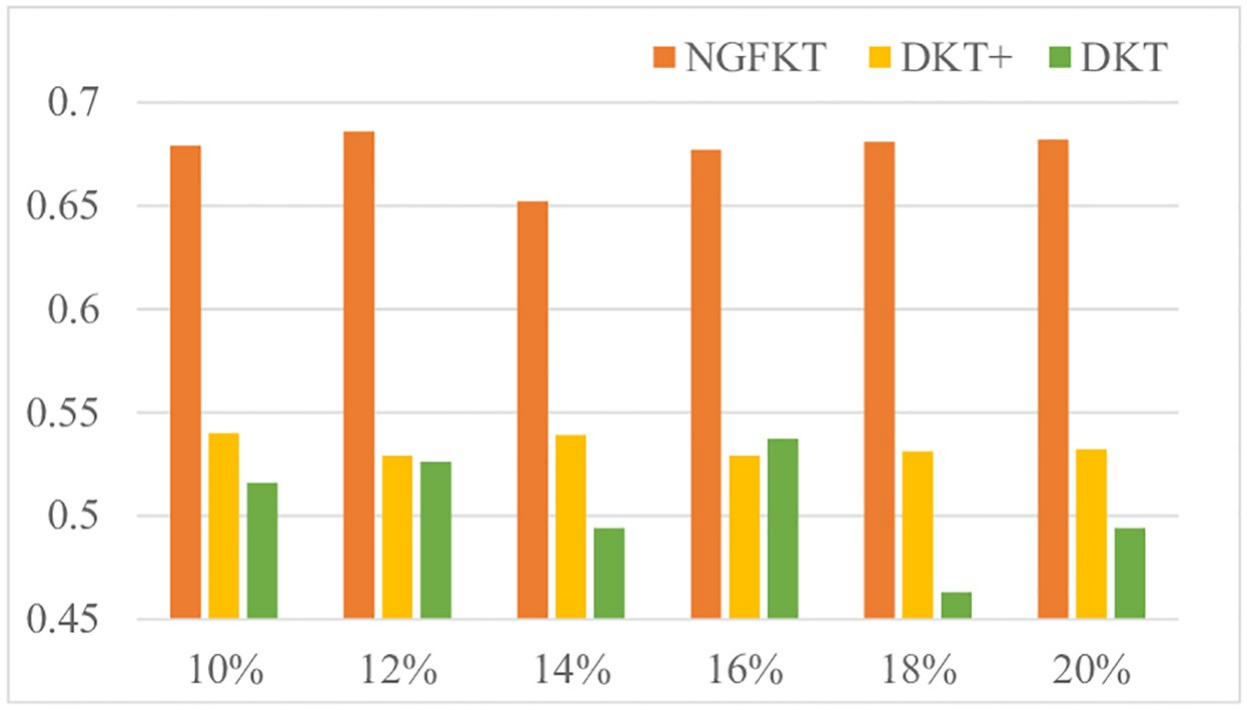
AUC on the Eedi dataset is compared when the number of the students is 10%, 12%, 14%, 16%, 18%, and 20% respectively. The Neural Graph Forgetting Knowledge Tracing Model outperforms the other two models.

In scenario 1, student populations ranging from 10% to 20% of the training dataset are tested to evaluate the prediction performance of NGFKT, DKT, and DKT+ referencing Fig 4. Specifically, the NFGKT model outperforms the DKT model and DKT+ model on the Eedi dataset because the NFGKT model considers the exercise relation modeling and applies the attention mechanism to process the data. And the accuracy of the NGFKT model remains at around 68%. In addition, the performance of the DKT model fluctuates relatively. Compared with the DKT model, the DKT+ model solves the reconstruction problem and the consistency problem to further improve the performance to predict the student knowledge state. The DKT+ model’s performance is still at about 53%.

**Fig 5:**
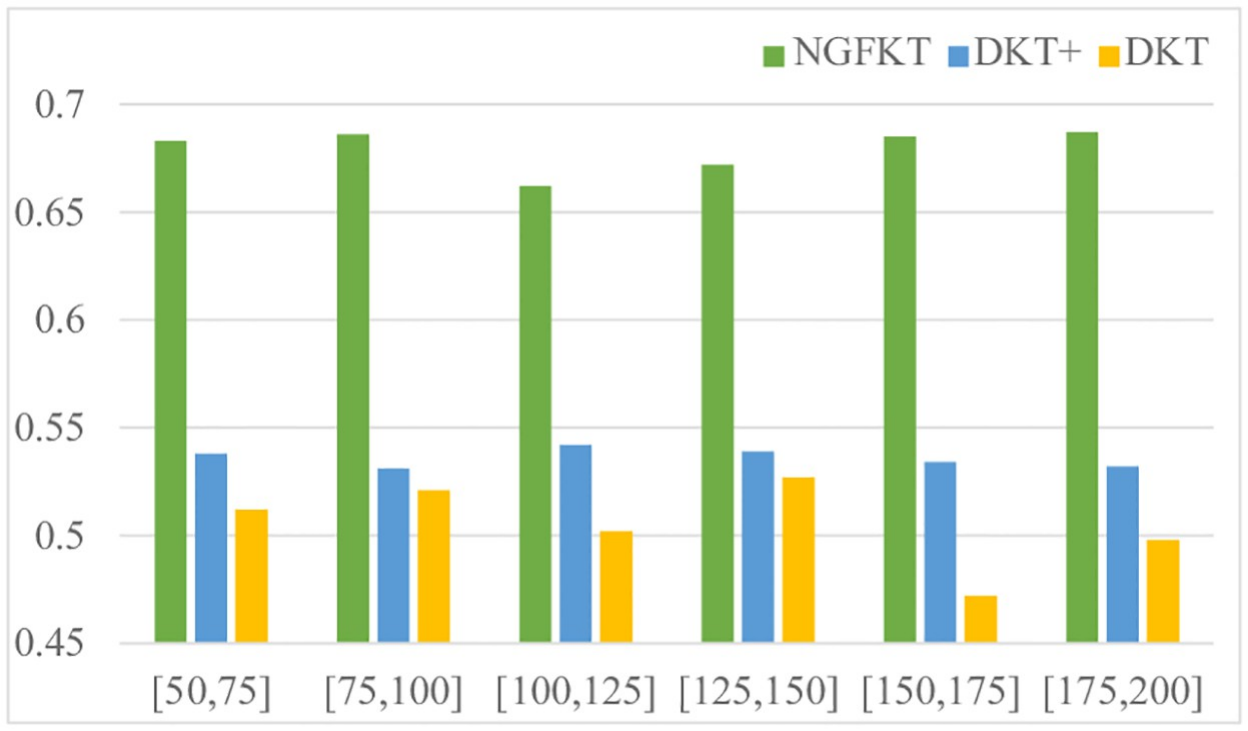
AUC on the Eedi dataset is compared when students are provided with 50, 75, 100, 125, 150, 175 and 200 answer records respectively. The Neural Graph Forgetting Knowledge Tracing Model achieves better results than the other two models.

In scenario 2, which contains new students who have short exercise sequences, the training data are separated into six groups. Each group has a distinct range of exercise sequences, such as (50, 75], (75, 100], (100, 125], (125, 150], (150, 175], and (175, 200]. The lengths of the exercises from the original exercise sequence are sampled to generate each exercise sequence in the training data. Given that students in the first group have the fewest exercise answering records, it is clear that this situation is the most challenging for the student. In Fig 5, the effectiveness of the various techniques is compared. On the Eedi datasets, the DKT+ model performs better than the DKT model by accepting two regularization items to enhance the model performance. However, the relation modeling part of the DKT+ model is ignored when the DKT+ model predicts the performance of students. Therefore, the NGFKT model solves this problem and shows better performance in this scenario than DKT and DKT+ by considering the relation modeling of the exercises and forgetting behavior of students.

#### Knowledge state prediction visualization

**Fig 6:**
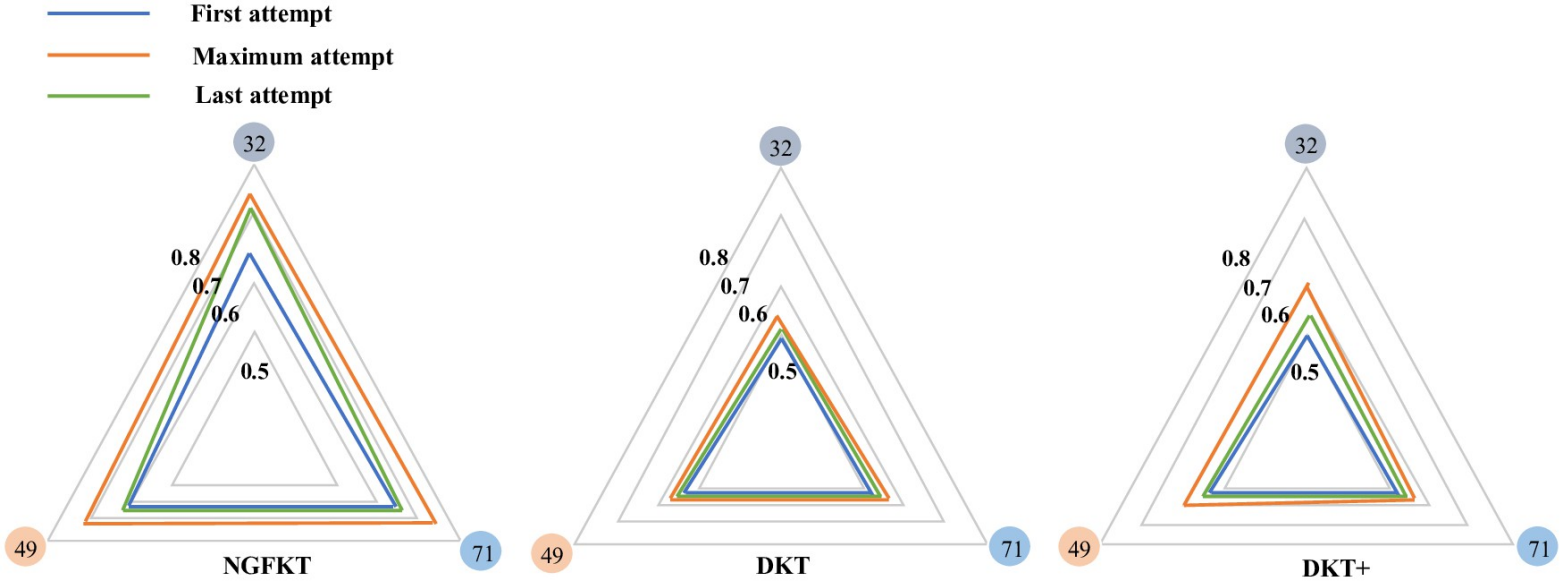
The radar diagram. The NGFKT model outperforms the DKT model and the DKT+ model in tracking the student knowledge state. The “32”, “49”, and “71” are three skill ids and are presented with three different colors. The average prediction accuracy of the NGFKT model is around 70.5%.

Knowledge state prediction visualization is regarded as an important application of knowledge tracing models for online educational systems. We will show that our proposed model: the NGFKT model can capture the student knowledge state correctly compared with two standard knowledge tracing models: the DKT model and the DKT+ model. Specifically, Fig 6 indicates the knowledge state traced by the NGFKT model of the same student. The general knowledge evolving process of the knowledge state is consistent with the student learning process. When the student first attempts the exercise, the knowledge state reaches the minimum level. The student continues to learn skills: “32”, “49”, and “71” and continuously deepens his proficiency in knowledge points. Finally, the student knowledge state achieves the maximum, which is shown by the increased areas of the radar diagram. During the latest attempt of the student, the knowledge proficiency of the student presents some reduction considering the student forgetting behavior. However, knowledge proficiency is still improved by continuously practicing the skills compared with the first interaction with the student.

Referring to Fig 6, the NGFKT model also outperforms the DKT model and the DKT+ model because the NGFKT model further incorporates the relation modeling that is generated by the GCN model. The DKT+ model achieves better results than the DKT model, which indicates that adding two regularization terms can further improve the performance of tracing the knowledge proficiency of the student.

## Conclusions and future work

In this paper, we introduced a novel knowledge tracing model, NGFKT, designed to accurately track students’ knowledge states by integrating relation modeling, skill relation matrices, Q-matrices, and relative distance representations. The NGFKT model effectively predicts students’ knowledge levels even with limited interaction data. Our approach employed the KRIRC method to calibrate skill relation matrices and Q-matrices, serving as inputs to a Graph Convolutional Network (GCN) for generating exercise and skill embeddings. By combining skill-exercise embeddings, item difficulty, and the contingency table, we derived the final exercise relation matrix. Employing the Position-Relation-Forgetting Attention mechanism yielded accurate predictions. Our experiments on two public datasets demonstrated the NGFKT model’s efficiency in tracking students’ knowledge states. The NGFKT model’s blend of explanatory and predictive power holds promise for enhancing the design of Online Educational Systems.

In this paper, we properly model the student, exercises, and skills to design an effective knowledge tracing model to estimate the knowledge state of students when considering the heterogeneous graph between them and the exercise difficulty. However, this paper still has two limitations of the knowledge tracing model. Firstly, the skill relation model should take exercise content into consideration to make the prediction more accurate. Secondly, other student behaviors such as guessing behavior or slipping behavior should be further discussed when the student factor is modeled.

In the future, we envision incorporating exercise texts into the knowledge tracing model’s design and integrating more nuanced student behaviors—such as the guessing and slipping factors—to enhance performance prediction. Additionally, we plan to expand the knowledge model’s scope to develop an innovative online intelligence system for recommending exercises to students. This system aims to create a seamless and tailored learning experience that adapts to individual student’s needs and preferences.
